# Molecular Simulation of the Isotropic-to-Nematic Transition of Rod-like Polymers in Bulk and Under Confinement

**DOI:** 10.3390/polym17121703

**Published:** 2025-06-19

**Authors:** Biao Yan, Daniel Martínez-Fernández, Katerina Foteinopoulou, Nikos Ch. Karayiannis

**Affiliations:** Institute for Optoelectronic Systems and Microtechnology (ISOM) and Escuela Técnica Superior de Ingenieros Industriales (ETSII), Universidad Politécnica de Madrid (UPM), José Gutierrez Abascal 2, 28006 Madrid, Spain; biao.yan@alumnos.upm.es (B.Y.); daniel.martinez.fernandez@upm.es (D.M.-F.); k.foteinopoulou@upm.es (K.F.)

**Keywords:** Monte Carlo, polymers, isotropic-to-nematic transition, phase behavior, hard sphere, nematic order, colloids, rod-like chains, confinement, semi-flexible, packing density, molecular simulation, chain stiffness

## Abstract

We conduct extensive Monte Carlo simulations to investigate the factors that control the isotropic-to-nematic transition of hard colloidal polymers in bulk and under various conditions of confinement. Utilizing a highly idealized model, polymers are represented as linear chains of tangent hard spheres of uniform length, whose stiffness is controlled by a bending potential leading to rod-like configurations. Confinement is realized through the presence of flat, parallel, and impenetrable walls in one, two, or three dimensions while periodic boundary conditions are applied on the unconstrained dimensions. All simulations are performed through the Simu-D software, composed of conventional and advanced, chain-connectivity-altering Monte Carlo algorithms. We explore in detail how distinct factors, including chain length, stiffness, confinement, and packing density affect the isotropic-to-nematic transition exhibited by the polymer chains and identify with high precision the concentration range where this phase change takes place as a function of the applied conditions.

## 1. Introduction

Synthetic polymers have an enormous number of applications in our daily life, including among others packaging, construction, clothing, fibers, paints, toys, coatings, optics, household items, insulation, medicine, and healthcare. For this reason, the connection between the behavior of atoms and of the salient characteristics of chain geometry and topology with macroscopic properties is a key element in the eventual design of polymer-based materials with tailored features. Towards this, elegant theories have been developed on the prediction of physical and chemical properties, including universal scaling laws of the static and dynamic behavior of polymers [1,2,3,4].

Nevertheless, polymer research can be quite challenging because of the wide range of characteristic time and length scales and the presence of entanglements which significantly constrain and reduce chain mobility [3,4,5]. Adding to this complexity, research, based on experiments or simulations, requires considering a plethora of chemical and physical factors and processing conditions. Moreover, macroscopic properties are also strongly dependent on the phase behavior of polymer systems, from the local intramolecular and intermolecular arrangement of the atoms to the global one of the chains. For example, liquid crystals, used in a wide range of applications and materials, show long-range orientational order which provides them with outstanding optical properties and stimuli-responsive behavior [6,7,8,9,10,11,12].

Liquid crystal (LC) is a unique state of matter exhibiting behavior that corresponds simultaneously to liquid and crystal, further showing varying levels of orientational and positional order depending on the conditions [13,14,15,16]. Onsager first predicted that thin, infinitely long and rigid hard particles, driven by entropy, transit from an isotropic phase to a nematic one when a certain concentration is reached [17]. Later, Bolhuis and Frenkel determined the phase diagram of the Onsager transition for hard spherocylinders as a function of shape anisotropy [18], while Khokhlov and Semenov established the dependence of this transition on chain size ratio for polymers [19,20,21]. As expected, for macromolecules in the rod limit, the isotropic-to-nematic transition occurs at a high concentration, though still lower than the corresponding one for solidification [22,23].

Molecular modeling in particular, and computer simulations in general [24,25,26,27,28], can provide significant insights into the mechanisms, at the level of atoms and molecules, that trigger phase behavior as exhibited by LC systems [29,30,31]. Furthermore, combined with refined descriptors to quantify local and global structure they can identify with very high precision established, thermodynamically stable morphologies [32,33,34,35,36,37]. Such a hierarchical, multiscale analysis is essential to understand phase transitions and general self-organization phenomena, which are the cornerstone characteristics of soft matter [38].

Employing Molecular Dynamics (MD) and Monte Carlo (MC) simulations, as well as efficient algorithms for the generation of such systems [39,40], independent authors have extensively researched the isotropic-to-nematic transition and the general phase behavior of semi-flexible polymers in bulk and under confinement in two and three dimensions [22,23,29,34,41,42,43,44,45,46,47,48,49,50,51,52,53,54,55,56,57,58,59]. Milchev et al. [41] studied the characteristics of I-N transitions in polymer blends with chains of different lengths and stiffness. With an increase of density, there is a gradual transition from the isotropic to the nematic phase, while chain length and stiffness affect the width of the mixing area. Milchev and Binder [42] used MD simulations to study phase behavior in semi-flexible polymer solutions in cylindrical pores with repulsive walls. Egorov et al. [47] employed density functional theory and MD simulations to study solutions of semi-flexible polymers constrained by repulsive planar walls, gauging the conditions under which the confining agents induce nematic order. Hoy and collaborators studied the effect of chain stiffness on the competition between glass transition and crystallization for model systems of hardsphere-like polymers under a variety of processing conditions through MD [50,51,60]. Chen and collaborators investigated phase transitions of semi-flexible polymers under confinement and in the presence of nanoparticles using Langevin dynamics [61,62,63].

At dilute conditions and for oligomers the isotropic-to-nematic transition of hard colloidal polymers can be studied by collision-driven MD simulations, suitably modified to respect the holonomic constraints imposed by chain connectivity. However, as concentration and/or chain length increase MD simulations are not able to provide equilibration, especially at the global level of chains, within reasonable computational time. Practically, the dynamics of such systems are too slow to be tracked efficiently by MD. On the other hand MC, by being a stochastic method, has specific advantages in the study of the phase behavior exhibited by polymers, including isotropic-to-nematic behavior, especially when chains of high molecular weight are involved at high concentrations [28,64,65,66,67,68,69]. Equilibration in MC simulations depends strongly on the efficiency of the involved algorithms (moves) [70]. As demonstrated in past studies, the combination of advanced, chain-connectivity-altering moves (like end-bringing [71,72] and double-bridging [73] for atomistic systems and simplified variants [33,74,75] for colloidal counterparts) and the implementation of a configurational bias scheme [76,77,78] for the execution of conventional moves is very efficient in providing full-scale equilibration even for volume fractions very close to the random close packed (RCP) limit of hard spheres [79,80].

Yethiraj and Cuetos [22,81,82] used MC to simulate the transition from isotropic to nematic states in semi-flexible polymers in the rod limit. Similarly, Bates et al. [83,84] studied phase transitions in liquid crystals with mesogens of different shapes. Egorov et al. [85] simulated and analyzed the shape and volume phase transitions of liquid crystal elastomers (LCEs) immersed in solvents using the Finsler geometry (FG) model. In other recent MC studies, Shakirov and Paul identified a first-order crystal transition in short, rod-like polymer melts [68,69]. In agreement with [68], our research group identified, through MC simulations, isotropic-to-nematic transition and crystallization in very dense packings composed of short, semi-flexible chains of tangent hard spheres [34]. According to the findings, nematic close-packed (NCP) structures are formed for rod-like chains at sufficiently high concentrations [50,51]. Thus, the 2D hexagonal crystal structures observed by Shakirov [68] evolve with increasing packing density into a 3D semi-crystalline phase and, eventually, form hexagonal close-packed (HCP), face-centered cubic (FCC) or mixed-ordered morphologies composed of both crystals. Interestingly, these NCP morphologies, formed at densities higher than the melting point for monomeric hard spheres, show order both at the local (spheres) and global (chains) level [34].

The main research objective of the present study is to gauge in detail the factors that affect the isotropic-to-nematic transition of linear, rod-like chains of tangent hard spheres. We practically expand our previous modeling studies on the local and global structure of fully flexible [75,80,86,87,88,89,90] and semi-flexible [34,52] polymers made of hard spheres in bulk [87] and under confinement [57,88,89], investigating the effect of factors like volume fraction [91], molecular length [74,79] and bond geometry [34,52,87]. We analyze the combined effect of the aforementioned factors on isotropic-to-nematic transition and identify with high precision the critical volume fraction where this takes place. For that, we perform extensive Monte Carlo simulations employing the Simu-D simulator-descriptor software [33] and quantify the global nematic order through the orientational order parameter, q [34,52]. Based on the present simulation findings, empirical equations are proposed to capture the effect of the processing conditions on isotropic-to-nematic transition.

This article is organized as follows: In Section 2, we will introduce the models and simulation methods employed here. The simulation results are presented and discussed in Section 3. Finally, Section 4 summarizes the main findings and conclusions.

## 2. Methodology

### 2.1. Molecular Model

Here we adopt a hard sphere colloidal model for polymer representation, as in our past studies [34,52,80,86,88,89,90]. Polymers are simulated as linear, rod-like chains of tangent spheres of uniform size, σ, which, further, is the characteristic length of the system. The chains have lengths (number of hard spheres), which fluctuate uniformly in the interval $N\in \left[N_{min},\;N_{max}\right]$, with the average chain length being $N_{av}$, as controlled by a properly selected spectrum of chemical potentials [74].

All non-bonded interactions are described by the hard sphere (HS) potential, prohibiting overlaps between sphere pairs [92]:

(1) $$U_{HS}(r_{ij})=\left\{\begin{array}{c} 0,\;\;\;\;\;r_{ij}\geq \sigma \\ \infty ,\;\;\;\;r_{ij}<\sigma \end{array}\right.$$

where $r_{ij}$ is the distance between the centers of the monomers i and j and $U_{HS}$ is the corresponding energy. The hard-core model [93,94,95] has been used extensively to study phase behavior and self-organization in ideal systems like spheres [96,97,98,99], disks [100,101], rods [81,102] and pentagons [103]. According to a very recent review by Royall et al. [104] the deceptively simple molecular HS model has been extensively utilized for experimental [105,106,107], theoretical [108,109,110,111], and numerical [112,113,114] research on colloidal systems at and beyond equilibrium [115].

**Figure 1:**
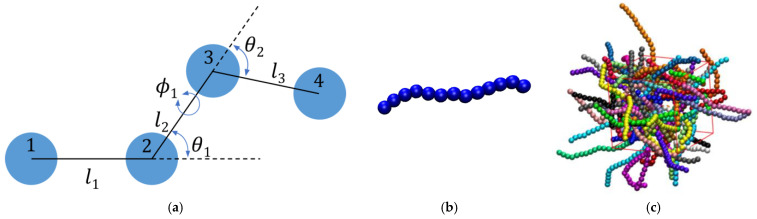
(**a**) Sketch of the bond geometry in a polymer chain, where l is the bond length between two successive atoms, θ is the bending angle between three successive atoms and ϕ is the torsion angle between four successive atoms. (**b**) A typical configuration of a rod-like chain being composed of 13 hard spheres. (**c**) Computer-generated configuration of a bulk ($d_{conf}=0$) system corresponding to $N_{av}=12$, $N_{ch}=100$, $k_{\theta }=9$ and φ=0.2. Monomers are colored according to the parent chains and are shown with their coordinates fully unwrapped in space. The red solid lines indicate the borders of the simulation cell. The snapshot images are generated using VMD software (version 1.9.3) [117].

Figure 1a shows the bond geometry in a polymer chain where l is the bond length, θ is the bending angle, and ϕ is the torsion angle. With respect to bond geometry, successive spheres along the chains are practically tangent within a numerical tolerance of $dl=6.5\times 10^{-4}$ (in units of σ). Panel (b) shows a typical conformation of a rod-like chain, while panel (c) shows computer-generated polymer packing under periodic boundary conditions. Chain stiffness is controlled through a harmonic potential on the bending angle θ. The bending potential is identical to the one used in our recent studies [34,52]:

(2) $$U_{bend}\left(\theta \right)=k_{\theta }{\left(\theta -\theta_{0}\right)}^{2}$$

where $k_{\theta }$ is the bending constant and $\theta_{0}$ is the equilibrium bending angle. For fixed bond lengths, setting $k_{\theta }=0$ or $k_{\theta }\to \infty$ allows the simulation of freely jointed or freely rotating chains, respectively. No potential is used here to control torsion angles, ϕ.

Concerning the simulation cell, four different cases are studied: (I) bulk, where periodic boundary conditions are applied on all dimensions ($d_{conf}=0$), (II) confinement in one dimension ($d_{conf}=1$), (III) confinement in two dimensions ($d_{conf}=2$), and (IV) full confinement ($d_{conf}=3$). As in our previous simulations confinement is enforced by the presence of flat, parallel, and impenetrable surfaces in at least one dimension [33,75,88,89]. In all cases, the simulation cell is cubic. When confinement is introduced, the inter-wall distance, $d_{wall}$ (in units of σ), is an important parameter to be considered with the limits $d_{wall}\;\to \;\sigma$ and $d_{wall}\;\to \;\infty$, corresponding to monolayer 2-D and bulk 3-D systems, respectively [52,57,75].

For non-overlapping objects, packing density (volume fraction), φ, is defined as the total volume occupied by the objects over the total volume of the simulation cell. In the case of hard spheres of uniform size, packing density is given by [34,116]:

(3) $$\varphi =\frac{V_{sph}}{V_{cell}}=\frac{\pi }{6}\frac{N_{at}}{V_{cell}}\sigma^{3}=\frac{\pi }{6}\frac{N_{av}N_{ch}}{V_{cell}}\sigma^{3}$$

where $N_{at}$ is the total number of hard spheres (monomers; $N_{at}=N_{ch}\;\cdot N_{av}$, $N_{ch}$ being the number of chains), $V_{sph}$ is the total volume occupied by the spheres, and $V_{cell}$ is the volume of the cubic simulation cell. For confined systems, given that the spheres cannot lie closer than σ/2 to each surface, an alternative definition of packing density, the effective packing density, $\varphi_{eff}$, can be introduced to account for the volume not accessible to the spheres, which additionally depends on $d_{conf}$. In general, for confined systems, $d_{wall}$ is the interwall distance along the confined dimension(s) and L is the length in the unconfined one(s), both measured in units of σ. If we consider that the length of the cell dimensions under the same conditions (periodic or confinement) is the same, we can modify Equation (3) as

(4) $$\varphi =\frac{\pi }{6}\frac{N_{at}}{{d_{wall}}^{d_{conf}}L^{\left(3-d_{conf}\right)}}\sigma^{3}$$

Setting $d_{conf}=0$ recovers the definition of the packing density for the bulk system as in Equation (3). Additionally, the effective packing density, $\varphi_{eff}$, can be expressed as [89]

(5) $$\varphi_{eff}=\frac{\pi }{6}\frac{N_{at}}{(d_{wall}-\sigma {)}^{d_{conf}}L^{\left(3-d_{conf}\right)}}\sigma^{3}$$

In Equations (4) and (5) for cubic cells the equality $L=d_{wall}$ is also valid.

Table 1 presents indicatively packing densities, φ, and effective counterparts, $\varphi_{eff}$, under different confinement conditions ($d_{conf}=0,\;1,\;2$ and 3) while keeping $d_{wall}$ constant (with the same $N_{ch}$ and φ). The $d_{wall}$ data are obtained from Table 2.

### 2.2. Simulation Algorithm

All simulations to be reported below are performed using the homemade Simu-D simulator-descriptor suite [33], using the same mix of MC moves as in our most recent works [34,52,90]. The MC scheme is composed of chain-connectivity-altering moves (simplified end bridging (sEB) and simplified intramolecular end bridging (sIEB)) and conventional ones (reptation, end-mer rotation, flip, intermolecular reptation, and end-group rotation) [33,74]. The latter are performed through a configurational bias scheme with the number of trial configurations depending strongly on packing density as described in [74]. The efficiency of the proposed MC method in generating and equilibrating polymer packing of very long chains at very high concentrations, even near and at the random close packed (RCP) limit of hard spheres, has been demonstrated in past publications [33,34,52,74,75,79,86,87,89].

The initial configurations of all systems are generated under very dilute conditions, at an initial packing density of φ=0.001. We consider as the reference system the one that corresponds to $N_{av}=12\;\left(N\in \left[6,\;18\right]\right),N_{ch}=100,\;N_{at}=1200,\;{\;d}_{conf}=0\;$and$\;k_{\theta }/k_{B}T=9\;{rad}^{-2}$ (for simplicity, the value of the bending constant will henceforth be presented as $k_{\theta }=9)$ as reported in [34]. Additionally, in all simulations we fix $T=1\;/\;k_{B}\;$and$\;\theta_{0}=0{}^{\circ}$. To study the effect of chain length the following cases are explored ${(d}_{conf}=0,\;k_{\theta }=9):$ $N_{av}=24,\;N_{ch}=50;\;N_{av}=100,\;N_{ch}=48;\;N_{av}=500,\;N_{ch}=20;\;N_{av}=1000,\;N_{ch}=10$. Similarly, to study the effect of chain stiffness the following systems have been simulated ($d_{conf}=0,\;N_{av}=12,\;N_{ch}=100,\;N_{at}=1200$): $k_{\theta }=0.1,\;0.5,\;1,\;2,\;4,\;9$ and 20.

**Figure 2:**
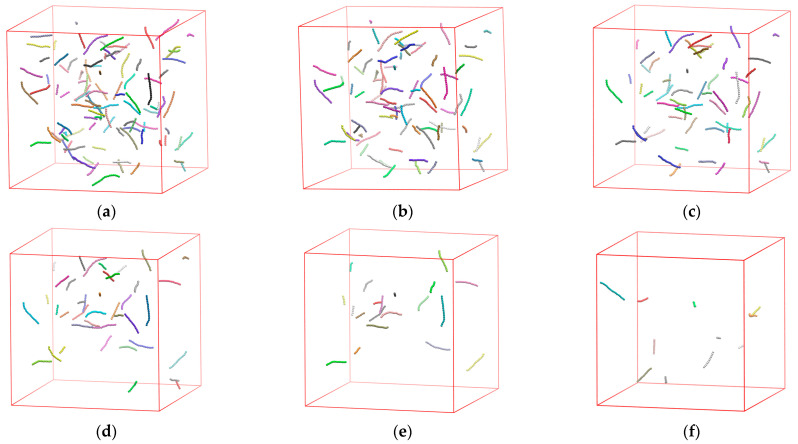
Series of snapshots illustrating the process of system generation with different $N_{ch}$ by randomly removing properly selected chains without altering the dimensions of the simulation cell or the average chain length. (**a**) Initial system ($N_{ch}=100,\;N_{at}=1200,\;\varphi =0.001$); (**b**) $N_{ch}=80,\;N_{at}=960,\;\varphi =0.0008$; (**c**) $N_{ch}=60,\;N_{at}=720,\;\varphi =0.0006$; (**d**) $N_{ch}=40,\;N_{at}=480,\;\varphi =0.0004$; (**e**) $N_{ch}=20,\;N_{at}=240,\;\varphi =0.0002$; (**f**) $N_{ch}=10,\;N_{at}=120,\;\varphi =0.0001$. All systems correspond to $N_{av}=12$, $k_{\theta }=9$ and $d_{conf}=0$. Monomers are colored according to the parent chain and are shown with the coordinates of their centers subjected to periodic boundary conditions. The snapshot images are generated using VMD software [117].

In order to obtain confined systems with varied inter-wall distances we start with $N_{av}=12$ and $N_{ch}=100$ in bulk at very dilute conditions, ensuring that chain connectivity is not broken due to periodic boundary conditions (panel (a) in Figure 2). We then proceed with the deletion of chains to generate configurations with $N_{ch}=80,\;60,\;40,\;20,\;$and 10, without altering cell dimensions or average chain lengths (panels (b–f) in Figure 2). Clearly, for a confined system, effective packing density ($\varphi_{eff}$) and inter-wall distance ($d_{wall}$) depend on volume fraction (φ), level of confinement ($d_{conf}$), and system size ($N_{at}$), or their combinations, as can be seen for example in Equation (4).

To obtain bulk or confined systems at higher volume fractions, the initial configurations are isotropically compressed through the Simu-D suite using the protocol described in [34,88] and respecting the applied boundary conditions (periodic or hard walls) until the desired density is reached.

### 2.3. Long-Range Order

In this study, we utilize the long-range orientational order, as presented in [31,34,52], to describe the spatial arrangement of polymer and to quantify long-range order. In the nematic phase, the alignment of the long axes of the chains exhibits a certain degree of directional consistency on a larger scale, referred to as the nematic director n. In contrast, in the isotropic phase, the orientations of the polymers are random. To quantify the degree of chain alignment, we numerically analyze the average orientation of the chains. Specifically, the orientation of each chain is represented by a unit vector u, which points along the long axis of the chain. The long axis of each chain is determined by the inertia tensor I. Equation (6) illustrates this second-order tensor.

(6) $$I=\sum_{i=1}^{N(j)}m_{i}\left(∥\frac{x_{i}-x_{cm}}{\sigma }{∥}^{2}\delta -x_{i}x_{i}\right)$$

Here, $N\left(j\right)$ is the number of monomers in chain j, $m_{i}$ and $x_{i}$ are the mass (assumed to be unity) and the coordinate vector, respectively, of monomer *i*, $x_{cm}$ is the coordinate vector of the center of mass of the chain, and δ is the second-order isotropic tensor. The eigenvector $v_{3}$, corresponding to the smallest eigenvalue of the inertia tensor I represents the orientation of the chain’s long axis. Finally, we normalize the vector $v_{3}$ to obtain the unit vector u.

(7) $$u=\frac{v_{3}}{∥v_{3}∥}$$

We define the second-order tensor Q as the average of the orientations of all the molecular chains, as follows:

(8) $$Q=\frac{1}{N_{ch}}\sum_{i=1}^{N_{ch}}u_{i}u_{i}-\frac{1}{3}\delta$$

The Q tensor can be used to describe the system in different ideal phases, such as the isotropic phase, prolate mesogen nematic mesophase, and oblate mesogen nematic mesophase [34]. For the “isotropic phase” (denoted as “ISO”), the orientations of the molecules are random, with equal probability for all directions, meaning that no preferred orientation exists. As a result, all components of the resulting $Q^{ISO}$ tensor are zero.

In the perfectly aligned prolate mesogen nematic mesophase, the long axes of the molecules are aligned in a specific direction, forming a nematic phase. In a coordinate system where the preferred direction of the system is along the *x*-axis, the matrix representation of the Q tensor for the ideal prolate mesogen nematic mesophase (denoted as “PRO”) is as follows:

(9) $$⟦Q^{PRO}⟧=\left(\begin{array}{ccc} \frac{2}{3} & 0 & 0\\ 0 & -\frac{1}{3} & 0\\ 0 & 0 & -\frac{1}{3} \end{array}\right)$$

In the oblate mesogen, nematic mesophase, the short axes of the molecules align with the nematic director n. In the same coordinate system, the Q tensor for the oblate mesogen, nematic mesophase, referred to as “OBL”, is represented as follows:

(10) $$⟦Q^{OBL}⟧=\left(\begin{array}{ccc} -\frac{1}{3} & 0 & 0\\ 0 & \frac{1}{6} & 0\\ 0 & 0 & \frac{1}{6} \end{array}\right)$$

To analyze the orientational characteristics of the system, we diagonalize the Q tensor and use its normalized eigenvectors as new coordinate axes, such that the transformed Q tensor, $Q^{\prime }$, becomes a diagonal matrix. The diagonal elements correspond to the eigenvalues $(\lambda_{1},\;\lambda_{2},\;$and$\;\lambda_{3})$, sorted in decreasing order of absolute value. In the nematic phase, the eigenvector corresponding to the largest absolute eigenvalue $\lambda_{1}$ represents the primary orientation of the system, i.e., the nematic director n.

To quantify the chain orientation order, we define a scalar order parameter q by comparing $⟦Q^{\prime }⟧$ with the matrix form of the ideal prolate mesogen nematic phase $⟦Q^{PRO}⟧$. This parameter is used to describe the degree of alignment of the molecular chains: in an isotropic system, where the Q tensor components are all nearly zero, q approaches 0; when all the molecules are aligned in the same direction, q=1, corresponding to a perfectly aligned prolate mesogen nematic phase; in the ideal oblate mesogen nematic phase, q=−0.5, thus the range of values for q is −0.5≤q≤1. Therefore, q can be used to quantitatively describe the orientational order of the system.

(11) $$⟦Q^{\prime }⟧=\left(\begin{array}{ccc} \lambda_{1} & 0 & 0\\ 0 & \lambda_{2} & 0\\ 0 & 0 & \lambda_{3} \end{array}\right)=q\left(\begin{array}{ccc} \frac{2}{3} & 0 & 0\\ 0 & -\frac{1}{3} & 0\\ 0 & 0 & -\frac{1}{3} \end{array}\right)\;\;\;\;\;\;\;\;\left|\lambda_{1}\right|>\left|\lambda_{2}\right|>\left|\lambda_{3}\right|$$

To summarize, we can characterize long-range orientational order using the scalar orientational (nematic) order parameter q and the nematic director n.

## 3. Results

**Figure 3:**
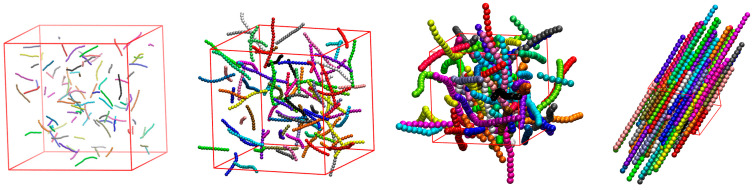
Snapshots of bulk ($d_{conf}=0$) systems corresponding to $N_{av}=12$, $N_{ch}=100$, and $k_{\theta }=9$. From left to right: φ=0.001, 0.01, 0.2, and 0.6. Spheres are colored according to the parent chain. Sphere coordinates are fully unwrapped in space. For visual clarity, the simulation cells are shown with similar dimensions, even if they correspond to very different volume fractions.

In the present study, we focus on the factors that affect the isotropic-to-nematic transition behavior and thus control the long-range order of athermal packings of semi-flexible polymers. This phase behavior is quantified by the nematic order parameter, q. In all systems studied, corresponding to different combinations of $N_{av}$, $d_{conf}$, $d_{wall},$ and $k_{\theta }$, we start from very dilute conditions (usually φ=0.001) and compress isotropically to generate systems of progressively higher concentrations. Figure 3 illustrates snapshots of bulk ($d_{conf}=0$) systems with $N_{av}=12$ and $k_{\theta }=9$ at packing densities of φ=0.001, 0.01, 0.2, and 0.6 with the corresponding nematic order parameters being equal to q=0.007, 0.008, 0.58, and 0.99, respectively.

### 3.1. Effect of Chain Length

**Figure 4:**
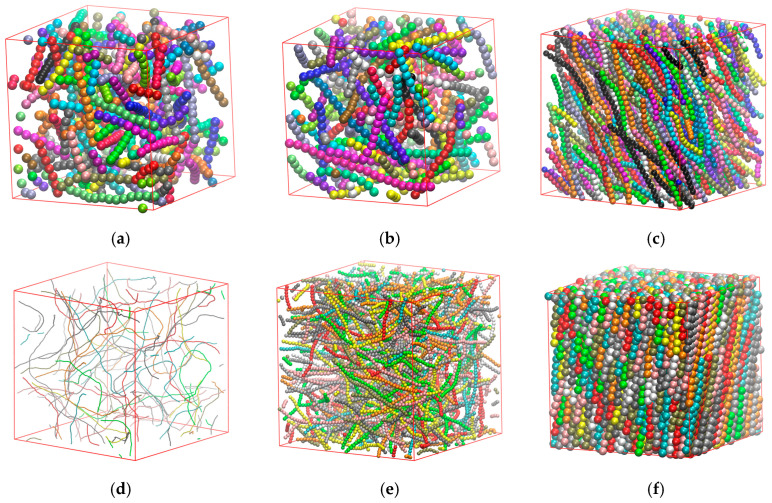
Snapshots of bulk ($d_{conf}=0$) systems corresponding to different combinations of $N_{av}$, $N_{at},$ and φ: (**a**) $N_{av}=12$, φ=0.1, $N_{at}=1200,$ and q=0.02; (**b**) $N_{av}=24$, φ=0.1, $N_{at}=1200,$ and q=0.11; (**c**) $N_{av}=100$, φ=0.1, $N_{at}=4800,$ and q=0.92; (**d**) $N_{av}=1000$, φ=0.001, $N_{at}=10,000,$ and q=0.08; (**e**) $N_{av}=1000$, φ=0.05, $N_{at}=10,000,$ and q=0.25; (**f**) $N_{av}=1000$, φ=0.58, $N_{at}=10,000,$ and q=0.99. In all cases $k_{\theta }=9$. Monomers are colored according to the parent chains and their coordinates are subjected to periodic boundary conditions. For visual clarity, the simulation cells are shown with approximately the same dimensions, even if they correspond to different volume fractions.

This section aims to investigate the effect of the average chain length, $N_{av}$, on isotropic-to-nematic transition in semi-flexible polymer systems in bulk ($d_{conf}=0$ and $k_{\theta }=9$). Figure 4 presents snapshots of semi-flexible polymer systems with different $N_{av}$ and φ.

**Figure 5:**
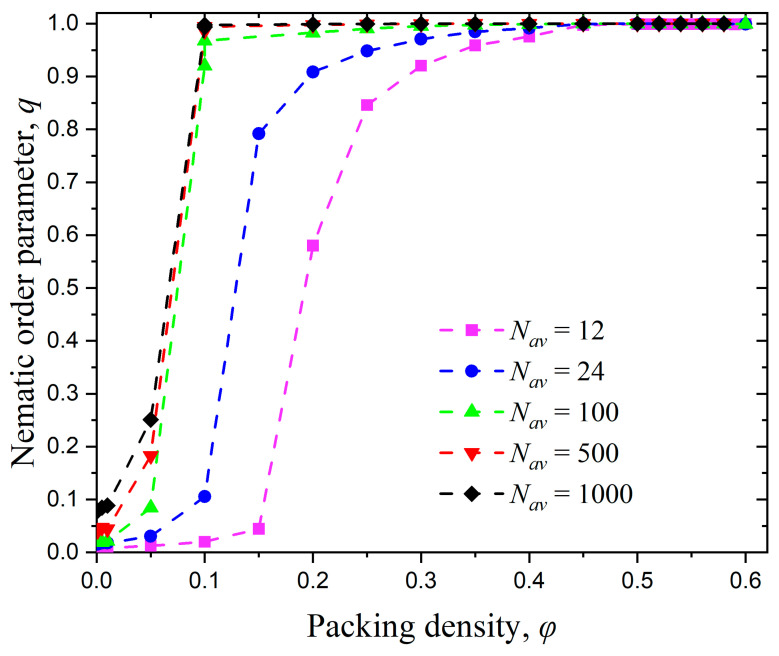
The evolution of the nematic order parameter, q, as a function of packing density, φ, versus average chain length, $N_{av},$ for bulk systems ($k_{\theta }=9$). Dashed lines connecting the simulation data serve only as guides for the eye.

Figure 5 shows the variation of the nematic order parameter, q, with the packing density, φ, for all $N_{av}$ bulk systems simulated here. At very dilute conditions, all systems remain in the isotropic state, with the nematic order parameter adopting low values, close to zero. As packing density increases, the nematic order parameter for all systems grows until it eventually reaches q=1 corresponding to a perfect nematic phase. By analyzing the established trends, we can observe that the systems of the shortest chain lengths $N_{av}=12$ and 24 show a more gradual increase of global order with packing density, while longer chains ($N_{av}=100$, 500, and 1000) exhibit a very abrupt change. Accordingly, longer molecular chains exhibit a shorter I-N transition process, which is further shifted to lower volume fractions. Midya et al. [118] studied polymers with chain lengths of N=8, 16, and 32 and found that the critical density decreases with increasing N, which is consistent with the simulation results presented in this section. Milchev et al. [45] reported an I-N transition point at 0.24 and 0.29 for N=32 and 64, respectively (in both cases $k_{\theta }=32$).

**Figure 6:**
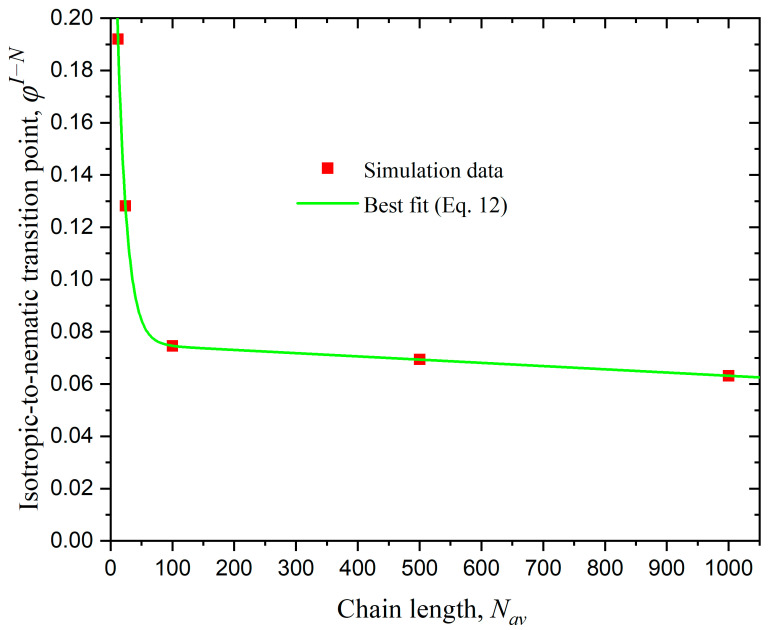
The isotropic-to-nematic transition point, $\varphi^{I-N}$, as a function of average chain length, $N_{av}$ for bulk systems of rod-like chains ($k_{\theta }=9$). The red points represent simulation results, while the green curve corresponds to the best fit according to the linear-exponential formula of Equation (12).

To further quantify the effect of the various factors on the phase behavior of rod-like athermal polymers, we identify the point of the I-N transition, $\varphi^{I-N}$, as the value of packing density above which the nematic order parameter becomes equal or higher to q ≥ 0.5. The red dots in Figure 6 demonstrate the variation pattern of the transition point at average chain lengths of $N_{av}=12,\;24,\;100,\;500,$ and 1000. From the figure, it is evident that the system with $N_{av}=1000$ undergoes the earliest transition ($\varphi^{I-N}\approx \;0.063$), whereas the system with $N_{av}=12$ exhibits the latest transition point ($\varphi^{I-N}\approx \;0.192$). Another interesting point is that $\varphi^{I-N}$ drops sharply for $N_{av}<100$ and the reduction is minimal for longer chains. To quantify the variation of $\varphi^{I-N}$ with $N_{av}$, we fitted the simulation data using the linear-exponential formula

(12) $$\varphi^{I-N}=a+b\;N_{av}+c\;r^{N_{av}}$$

where a=0.076, $b=-1.236\times 10^{-5}$, c=0.257, and r=0.936 (reliability coefficient $R^{2}=1$) for the bulk systems studied here ($d_{conf}=0$ and $k_{\theta }=9$).

### 3.2. Effect of Chain Stiffness

**Figure 7:**
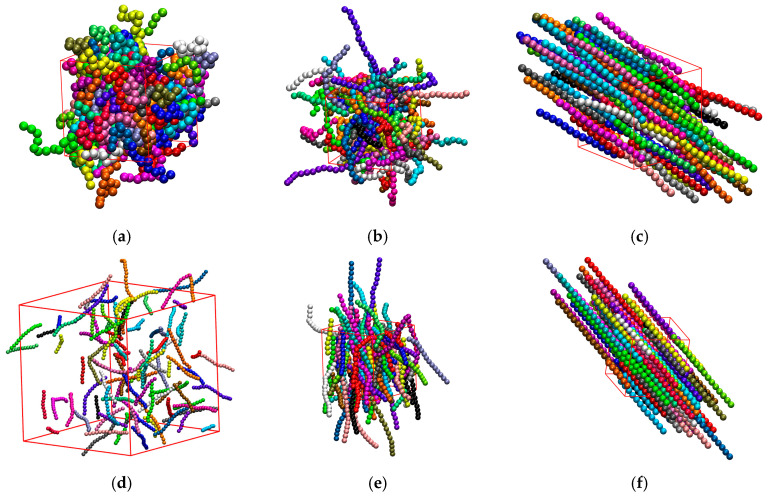
Snapshots of bulk systems of rod-like hard sphere chains with $N_{ch}=100$ and $N_{av}=12$ and different $k_{\theta }$ and φ. (**a**) $k_{\theta }=0.1$, φ=0.4, and q=0.01; (**b**) $k_{\theta }=1$, φ=0.4, and q=0.88; (**c**) $k_{\theta }=20$, φ=0.4, and q=0.98; (**d**) $k_{\theta }=4$, φ=0.01, and q=0.006; (**e**) $k_{\theta }=4$, φ=0.25, and q=0.69; (**f**) $k_{\theta }=4$, φ=0.6, and q=0.99.

This section aims to investigate the effect of the bending constant, $k_{\theta }$, on the isotropic-to-nematic transition in semi-flexible polymer systems in the bulk (in all cases $N_{av}=12$). Figure 7 presents snapshots of semi-flexible polymer systems with different $k_{\theta }$ and φ.

**Figure 8:**
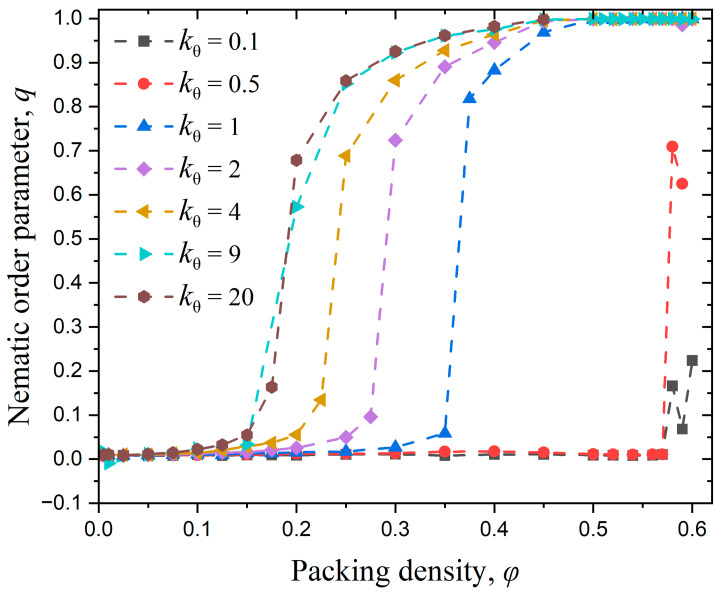
Evolution of the nematic order parameter, q, as a function of packing density, φ, for different values of bending constant, $k_{\theta }$. In all simulated systems $N_{av}=12$, $N_{ch}=100,$ and $d_{conf}=0$. Dashed lines connecting the simulation data serve only as guides for the eye.

Figure 8 illustrates the variation of the nematic order parameter, q, with the packing density, φ, for different values of bending stiffness, $k_{\theta }$. As the packing density increases, all systems undergo a complete isotropic-to-nematic transition, except the system of $k_{\theta }$ =0.1. The latter system does not reach a nematic order parameter higher than 0.25 for the whole concentration range. As a rule, the stronger the employed bending potential is the sooner the isotropic-to-nematic transition takes place. Luzhbin [63] compared the I-N transition in semiflexible polymer systems with $k_{\theta }=10,\;20$, and 100 and a fixed chain length of N=25, and found that the I-N transition point decreases with increasing $k_{\theta }$, which is consistent with the trend observed in this study. Milchev [45] investigated polymer systems with the same chain length but different bending stiffness values of $k_{\theta }=16,\;32$, and 64, and found the corresponding transition densities to be 0.43, 0.29, and 0.22, respectively. The study concluded that an increase in $k_{\theta }$ accelerates the I-N transition as also observed here.

The system of $k_{\theta }=0.5$ shows a significant delay as the phase change to nematic ordering occurs in a density range which practically coincides with the melting transition for fully flexible polymers of tangent hard spheres [86,87]. Because of this and as the volume fraction is very close to the random close packing (RCP) limit for rod-like chains with $k_{\theta }=9$, located at $\varphi^{RCP}\left(N_{av}=12\right)\;\approx 0.61$ according to [119], the established nematic phase for $k_{\theta }=0.5$ is defect ridden, in sharp contrast to the behavior of systems with higher bending stiffness.

**Figure 9:**
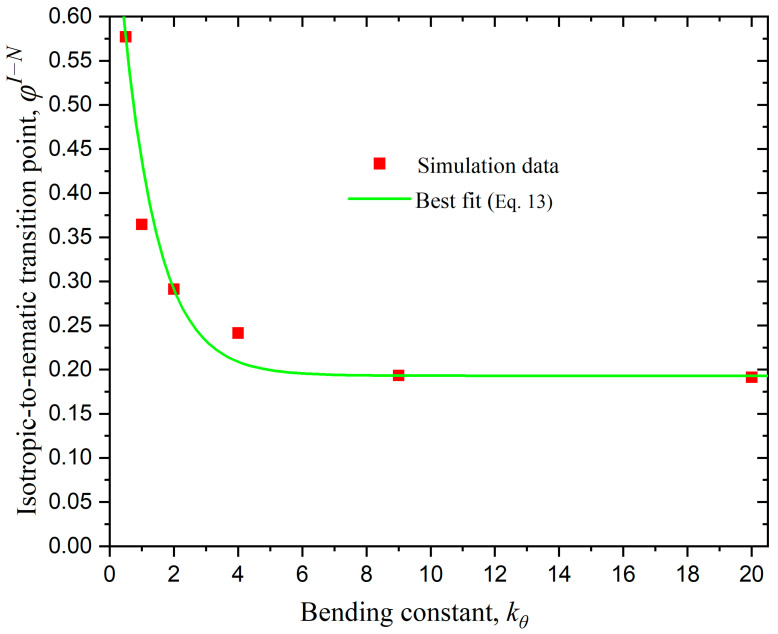
The isotropic-to-nematic transition point, $\varphi^{I-N},$ as a function of bending constant, $k_{\theta },$ for bulk systems of rod-like chains ($N_{av}=12$, $N_{ch}=100$). The red points represent simulation results, while the green curve corresponds to the best fit according to the formula of Equation (13).

Figure 9 shows the evolution of the isotropic-to-nematic transition point with the intensity of the bending potential $k_{\theta }$. We fit the transition point data $\varphi^{I-N}$ with the following formula of exponential decay:

(13) $$\varphi^{I-N}=a\;e^{b\;k_{\theta }}+c$$

where a=0.605, b=−0.91, and c=0.193. The reliability coefficient of the fit is $R^{2}=0.941$. Based on simulation results, initially there is a strong dependence on chain stiffness and the nematic transition shifts to lower concentrations. However, after a critical value is reached (which is estimated as $k_{\theta }\geq \;9$) there is no change in the isotropic-to-nematic transition point. As stated earlier, the system with $k_{\theta }=0.1$ is the only one that does not exhibit an isotropic-to-nematic transition. Therefore, we can conclude that the value of $k_{\theta }=0.5$ is the lowest, among the ones employed here, that guarantees an isotropic-to-nematic transition when fixing $N_{av}=12$ and $d_{conf}=0$ (bulk case).

### 3.3. Effect of Confinement

This study aims to examine the effect of spatial confinement on isotropic-to-nematic transition in semi-flexible polymer systems. Under bulk conditions, the system extends infinitely in all three dimensions and if it is sufficiently large the effect of $N_{at}$, $N_{ch},$ and L is not relevant. However, when the system is confined in at least one dimension, the variation in the parameters listed above, further including the one of $d_{wall}$, becomes a critical factor influencing phase behavior. As stated in the methodology section, to study the effect of the intensity of confinement, quantified here through $d_{wall}$, we generate systems with different dimensions under constant density. This is achieved by removing chains from the reference system ($N_{ch}=100$), while we keep the average length constant ($N_{av}=12$). $d_{wall}$ reflects accurately the intensity of confinement for a fixed volume fraction but it decreases when the concentration increases. Consequently, below we investigate the intensity of confinement through the relative chain number, defined simply as $x_{i}=N_{ch}(i)/N_{ch}(ref)$, where i is the index of the confined system, being composed of $N_{ch}(i)$ while $N_{ch}(ref)$, corresponds to the number of chains in the reference system, here being equal to 100. x represents the reduction in the number of chains relative to the initial system on a global scale.

Confined systems with different chain numbers ($N_{ch}=100,\;80,\;60,\;40,\;20,\;$and 10) are created based on the original bulk system ($N_{ch}=100$), while all other parameters remain unchanged, corresponding to relative chain numbers of x=1, 0.8, 0.6, 0.4, 0.2, and 0.1, respectively. To more accurately reflect the packing density under spatial confinement, in this section, we replace packing density, φ, with the effective packing density, $\varphi_{eff}$.

#### 3.3.1. Effect of Confinement in One Dimension

**Figure 10:**
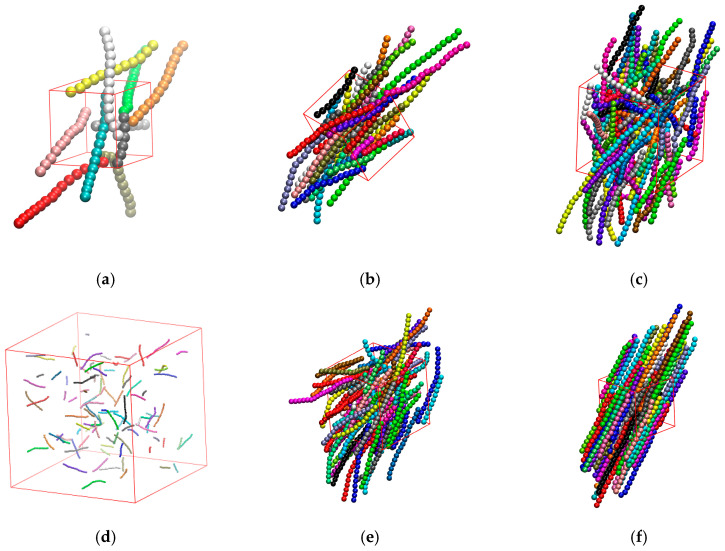
Snapshots of semi-flexible polymer systems under confinement in one dimension ($d_{conf}=1$), with different x and $\varphi_{eff}$. (**a**) x=0.1, φ=0.15 ($\varphi_{eff}=0.173$), and q=0.52; (**b**) x=0.4, φ=0.15 ($\varphi_{eff}=0.164$), and q=0.30; (**c**) x=1, φ=0.15 ($\varphi_{eff}=0.16$), and q=0.12; (**d**) x=1, φ=0.001 ($\varphi_{eff}=0.001$), and q=0.008; (**e**) x=1, φ=0.2 ($\varphi_{eff}=0.215$), and q=0.66; (**f**) x=1, φ=0.6 ($\varphi_{eff}=0.666$), and q=0.99. For all systems, $N_{av}=12$ and $k_{\theta }=9$.

Figure 10 presents snapshots of systems with different $N_{ch}$ values and effective packing densities, $\varphi_{eff}$, under confinement in one dimension ($d_{conf}=1$).

**Figure 11:**
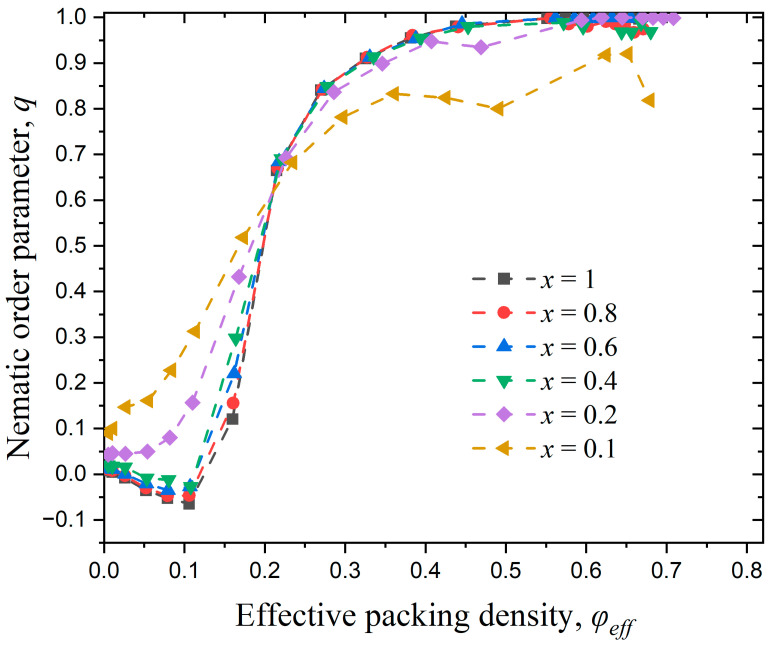
Evolution of the nematic order parameter, q, as a function of effective packing density, $\varphi_{eff}$, for systems with different relative chain numbers, x. For all systems $N_{av}=12$, $k_{\theta }=9$ and $d_{conf}=1$. Dashed lines connecting the simulation data serve only as guides for the eye.

Figure 11 illustrates the variation of the nematic order parameter, q, with the effective packing density, $\varphi_{eff}$, in semi-flexible polymer systems under confinement in one dimension, with x=1, 0.8, 0.6, 0.4, 0.2, and 0.1. According to the data presented in the figure, all systems show an isotropic-to-nematic transition once a critical effective concentration is reached. The most confined system (x=0.1) is the one that transits the earliest, i.e., at the lowest effective density; however, it is also the only system where imperfect nematic ordering (q<0.9) is established. Similar trends are exhibited for x=0.2, while the phase behaviors of all other systems are almost identical, showing perfect nematic ordering once a critical volume fraction is reached.

#### 3.3.2. Effect of Confinement in Two Dimensions

**Figure 12:**
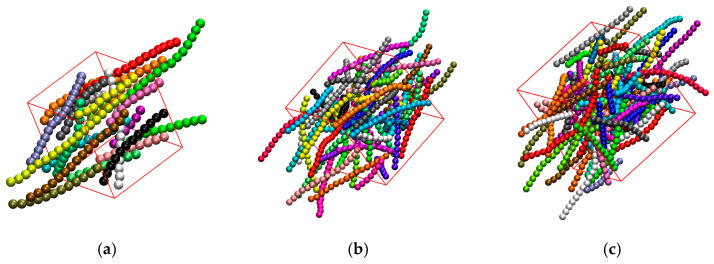

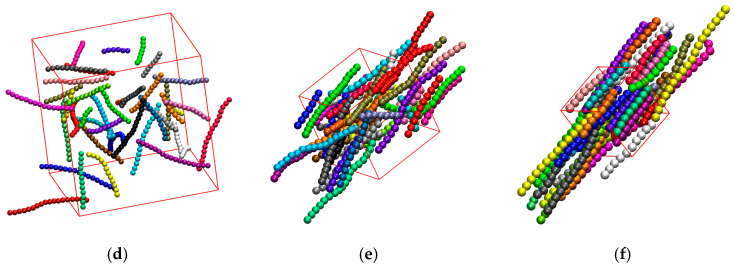
Snapshots of semi-flexible polymer systems under two-dimensional confinement ($d_{conf}=2$), with different x and $\varphi_{eff}$. (**a**) x=0.2, φ=0.1 ($\varphi_{eff}=0.121$), and q=0.67; (**b**) x=0.6, φ=0.1 ($\varphi_{eff}=0.114$), and q=0.46; (**c**) x=0.8, φ=0.1 ($\varphi_{eff}=0.113$), and q=0.40; (**d**) x=0.4, φ=0.01 ($\varphi_{eff}=0.011$), and q=0.04; (**e**) x=0.4, φ=0.15 ($\varphi_{eff}=0.179$), and q=0.75; (**f**) x=0.4, φ=0.54 ($\varphi_{eff}=0.712$), and q=0.99. For all systems $N_{av}=12$, $k_{\theta }=9$.

This section aims to investigate the isotropic-to-nematic transition in semi-flexible polymer systems under confinement in two dimensions ($d_{conf}=2$). We follow the same methodology and study the same systems as in the previous section, the only difference being that now the impenetrable flat walls are applied on two dimensions. Figure 12 presents snapshots of confined systems characterized by different x and $\varphi_{eff}$ values.

**Figure 13:**
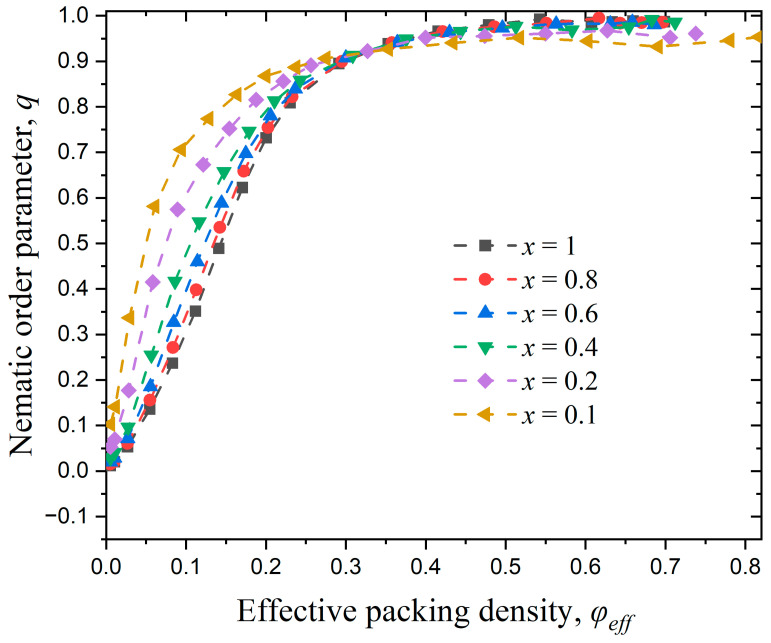
Evolution of the nematic order parameter, q, as a function of effective packing density, $\varphi_{eff}$, for different relative numbers of chains, x. For all systems $N_{av}=12$, $k_{\theta }=9,$ and $d_{conf}=2$. Dashed lines connecting the simulation data serve only as guides for the eye.

Figure 13 illustrates the variation of the nematic order parameter, q, with effective packing density, $\varphi_{eff}$, in semi-flexible polymer systems under confinement in two dimensions. The general tendency observed for $d_{conf}=2$ is very similar to the behavior exhibited for $d_{conf}=1$. The most confined systems (x=0.1 and 0.2) are the ones that transit to the nematic phase the soonest, but also are the systems that show the least perfect long-range ordering.

**Figure 14:**
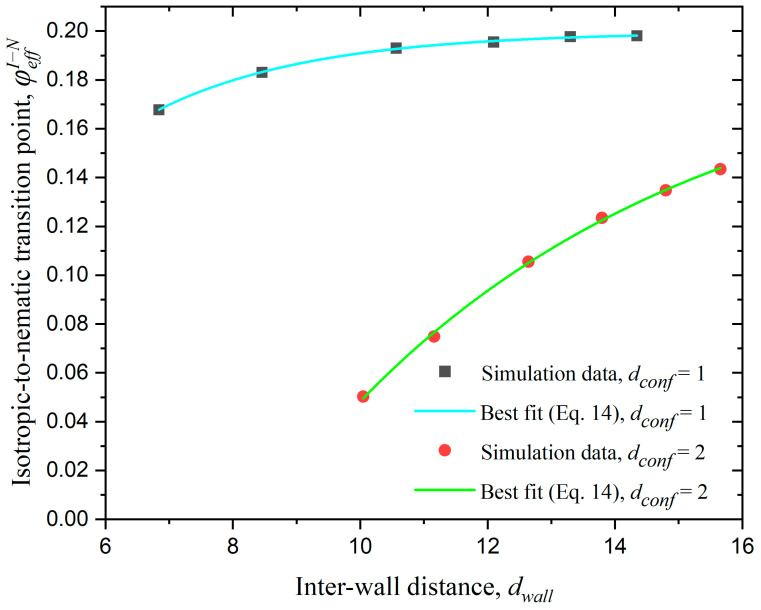
The isotropic-to-nematic transition point, $\varphi_{eff}^{I-N},$ as a function of inter-wall distance, $d_{wall}$ for confinement in one ($d_{conf}=1$) and two ($d_{conf}=2$) dimensions ($N_{av}=12$, $k_{\theta }=9$). The red and black points represent simulation results, while the green and cyan curves correspond to best fits according to the asympotic formula of Equation (14).

Figure 14 shows the isotropic-to-nematic transition point, $\varphi_{eff}^{I-N}$ as a function of $d_{wall}$ for the systems under confinement in one and two dimensions. $d_{wall}$ is calculated through Equation (5) since $\varphi_{eff}$, $N_{ch}$ (or x) and, accordingly, $N_{at}$ are known for each system under study. Comparing the data in Figure 11, Figure 13 and Figure 14, we can conclude that as confinement becomes stronger the isotropic-to-nematic transition occurs at lower concentrations and the final chain morphologies show imperfections with respect to the ideal nematic state. Egorov et al. [47,49] conducted simulations on semiflexible polymer systems under confinement and found that the presence of walls can induce nematic ordering, and this effect becomes more pronounced with increasing confinement. This is consistent with the findings of the present study.

In both cases of confinement, we fit the $\varphi_{eff}^{I-N}\;vs.\;d_{wall}$ simulation data with asymptotic formulas:

(14) $$\varphi_{eff}^{I-N}=a-b\;c^{d_{wall}}$$

where a=0.2, b=0.523, and c=0.664 for $d_{conf}=1$ and a=0.198, b=0.911 and c=0.835 for $d_{conf}=2$. In both cases an excellent agreement is observed between the asymptotic formula and the simulation data, as demonstrated by the reliability coefficient $R^{2}=1$. The bulk case ($d_{conf}=0$) is the limit where the interwall distance becomes infinitely large, i.e., $d_{wall}\;\to \;\infty$, so that the effect of confinement is practically eliminated. Extrapolating, according to the fitting data based on Equation (14), we find values of $\varphi_{eff}^{I-N}(d_{wall}\to \infty )=0.2$ and 0.198 for confinement in one and two dimensions, respectively. These values are in excellent agreement with the value of $\varphi^{I-N}=0.192\;$for $N_{av}=12$ system in the bulk, as seen in the simulation data in Figure 6.

#### 3.3.3. Effect of Confinement in Three Dimensions

**Figure 15:**
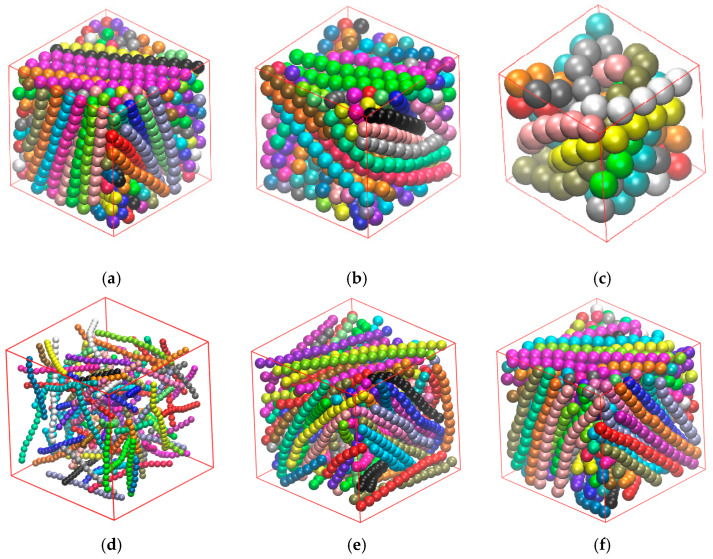
Snapshots with different x and $\varphi_{eff}$ for systems under full confinement ($d_{conf}=3$). (**a**) x=1, φ=0.4 ($\varphi_{eff}=0.524$), and q=0.086; (**b**) x=0.6, φ=0.4 ($\varphi_{eff}=0.552$), and q=0.001; (**c**) x=0.1, φ=0.4 ($\varphi_{eff}=0.74$), and q=0.117; (**d**) x=1, φ=0.05 ($\varphi_{eff}=0.057$), and q=0.013; (**e**) x=1, φ=0.2 ($\varphi_{eff}=0.247$), and q=−0.005; (**f**) x=1, φ=0.35 ($\varphi_{eff}=0.453$), and q=0.094. In all cases, $N_{av}=12$ and $k_{\theta }=9$.

This section focuses on the effect of full confinement ($d_{conf}=3$) on the phase behavior of athermal rod-like polymers. Figure 15 presents snapshots at the same packing density φ=0.4 for x=1, 0.6, and 0.4 and snapshots with the same x value (x=1) for φ=0.05, 0.2, and 0.35 under full confinement. Visual inspection suggests that the systems lack nematic ordering at the global scale. Furthermore, under strong confinement (x=0.1), chains are forced to curve and adopt configurations that are not compatible with the employed bending potential, which strongly favors straight configurations for successive triplets. This is not surprising, as the average length of the chains, as quantified by the end-to-end distance, $R_{ee}$, becomes commensurate to the inter-wall distance. In the ideal case of fully extended conformations, the maximum length, which corresponds to $N_{max}=18$, adopts the value of $R_{ee}^{ideal}(N_{max})=17$, if we consider the distance between the centers of the chain ends, or 18 if we further take into account the extra contour due to the sphere radius in both ends of the polymer.

To understand the long-range packing frustration, introduced by full confinement, in Table 3 we compare the average size of chains with $\sqrt{\langle R_{ee}^{2}\rangle }(N_{max})$, as obtained from simulations for selected systems in bulk and under full confinement. Also reported are the values of the inter-wall distance, $d_{wall}$. It can be observed that at low density (φ=0.05) the average dimensions for the longest chains are very similar and independent of the level of confinement. However, as the concentration and/or the intensity of confinement increase a significant deviation is observed: the fully confined chains adopt more compact conformations as they simply cannot fit otherwise in the simulation cell. Such coiling disallows the adaptation of extended, rod-like conformations. Accordingly, it is expected that such chain systems are not able to show nematic ordering as is already clear from the visual inspection of panels in Figure 15.

**Figure 16:**
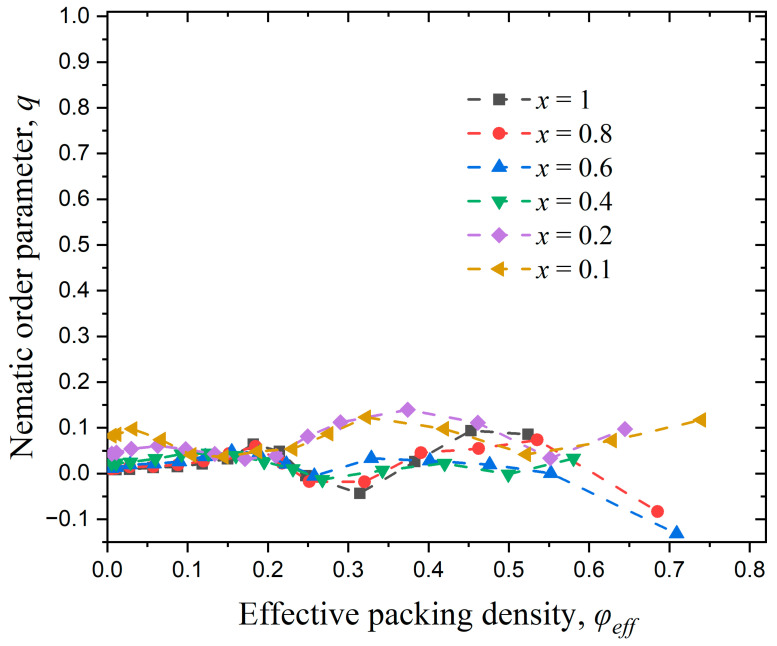
Evolution of the nematic order parameter, q, as a function of effective packing density, $\varphi_{eff}$, for different numbers of chains, $N_{ch}$ under full confinement. In all cases $N_{av}=12$ and $k_{\theta }=9$. Dashed lines connecting the simulation data serve only as guides for the eye.

Figure 16 illustrates the nematic order parameter as a function of the effective packing density for semi-flexible polymer systems with $N_{av}=12$ and $k_{\theta }=9$ under full confinement ($d_{conf}=3$). As can be seen from the graph, the nematic order parameter oscillates around very low positive and negative values, indicative of isotropic, long-range behavior. Analyzing the results for confinement in one (Figure 11) and two (Figure 13) dimensions we can conclude that the lowest value of the effective volume fraction where nematic ordering takes place corresponds to $\varphi_{eff}\approx 0.05$. At such concentrations the fully confined system exhibits a complete absence of long-range order, as quantified by q≈0.013 (Figure 16 and Table 3), even if the chain dimensions for the longer confined chains are on average very similar to the ones exhibited by the polymers in the bulk.

This behavior demonstrates that full spatial confinement inhibits nematic ordering by causing packing frustration at the level of chains. In a fully confined system, as the inter-wall distance decreases, polymers are forced to bend, losing their rod-like conformations, effectively inhibiting long-range orientational ordering.

## 4. Conclusions

In this study, we performed extensive Monte Carlo simulations to study the long-range phase behavior of linear, rod-like polymers made of tangent hard spheres of uniform size. We studied systematically how the packing density, average lengths of chains, $N_{av}$, the bending constant, $k_{\theta }$ and the level and intensity of confinement affect the isotropic-to-nematic transition. In addition, when such a change takes place, we identify with precision the corresponding volume fraction as a function of the aforementioned factors. In all cases, the global structure of the computer-generated system configurations is quantified by the nematic order parameter, q. The main objective of the research is to identify means to control effectively the final morphology of hard colloidal polymers.

Specifically, increasing the average chain length in a semi-flexible polymer system reduces the critical packing density needed for the isotropic-to-nematic transition to occur. At the same time, systems with $N_{av}\;\geq 100$ show polymeric behavior, as the isotropic-to-nematic transition point is not affected by a further increase in chain length. Chain stiffness affects profoundly the isotropic-to-nematic transition as a stronger bending potential shifts the transition to significantly lower concentrations. For the rod-like athermal polymers studied here, a critical value of $k_{\theta }=0.5$ or higher is required for the phase change to happen.

Finally, the level and intensity of confinement strongly affect the ability of chains to transit to nematic order. In general, for a given level of confinement (in one or two dimensions) the stronger the confinement, as quantified by the interwall distance, the earlier the isotropic-to-nematic transition takes place. However, for the strongest cases of confinement the corresponding systems are not able to reach the same levels of nematic perfection as the ones of weaker confinement. Finally, full confinement inhibits completely the long-range ordering transition in all simulated cases.

Attempting a comparison with experimental realizations, isotropic-to-nematic transition has been observed in independent studies on synthetic and natural clay rods [120,121], liquid-crystal droplets [122], mixtures of charged semiflexible rods and neutral polymers [123], suspensions of hard rod-like colloidal particles [124], and colloidal rods under confinement [125]. For example, Klop et al. [125] investigated the nematic ordering of colloidal rod-like particles under confinement and found that stronger confinement led to more regular alignment of the rods and higher degrees of nematic ordering. DFT simulation results showed similar trends. Maeda et al. [124] used optical microscopy to observe the arrangement of rod-like colloidal particles in suspension and found that rods with an aspect ratio of 10–35 (longer chains) more readily formed nematic phases compared to those with an aspect ratio of 3.5–8.0 (shorter chains). Dogic et al. [123] explored the phase behavior of mixtures of charged semiflexible rod-like viruses and neutral polymers, finding that increased chain stiffness facilitated transition to the nematic phase. Further experimental works investigate the behavior of granular and colloidal polymers and monomers under various conditions, including the effect of confinement [126,127,128,129,130,131,132,133].

The present study is currently being extended to treat semi-flexible chains under cylindrical and spherical confinement.

## Figures and Tables

**Table 1:** The correspondence between packing density, φ and effective packing density, $\varphi_{eff}$, as well as the inter-wall distance, $d_{wall}$, and cell dimension, L, for selected systems of $N_{ch}=100,\;k_{\theta }=9,\;N_{av}=12$ in $d_{conf}=0,\;1,\;2\;and\;3$.

φ ($d_{conf}=0$)	0.1	0.2	0.3	0.4	0.5
$\varphi_{eff}$ ($d_{conf}=1$)	0.106	0.215	0.325	0.438	0.551
$\varphi_{eff}$ ($d_{conf}=2$)	0.112	0.23	0.353	0.479	0.607
$\varphi_{eff}$ ($d_{conf}=3$)	0.118	0.247	0.383	0.524	0.669
$d_{wall}$ or L	18.45	14.64	12.79	11.62	10.79

**Table 2:** The relationship between cell dimension, L, and packing density, φ, in systems with different number of chains, $N_{ch}$, under periodic boundary conditions (bulk case).

φ	0.005	0.1	0.2	0.3	0.4	0.5
L ($N_{ch}=100$)	50.08	18.45	14.64	12.79	11.62	10.79
L ($N_{ch}=80$)	46.49	17.13	13.59	11.88	10.79	10.01
L ($N_{ch}=60$)	42.24	15.56	12.35	10.79	10.01	9.80
L ($N_{ch}=40$)	36.90	13.59	10.79	9.42	8.56	7.95
L ($N_{ch}=20$)	29.29	10.79	8.56	7.48	6.79	6.31
L ($N_{ch}=10$)	23.25	8.56	6.79	5.94	5.39	5.01

**Table 3:** Packing density, φ, effective packing density, $\varphi_{eff}$, relative chain number, x, inter-wall distance $d_{wall}$, and the square root of the mean square end-to-end distance as obtained from the simulations for the chains of maximum length ($N_{max}=18$), $\sqrt{\langle R_{ee}^{2}\rangle }\left(N_{max}\right)$, for the systems shown in Figure 15. Also shown for comparison are the corresponding simulation data for the bulk case ($d_{conf}=0$). For the latter, case numbers in parentheses correspond to packing density.

Case	φ	$\varphi_{eff}$	x	$d_{wall}$	$\sqrt{\langle R_{ee}^{2}\rangle }(N_{max})$	$\sqrt{\langle R_{ee}^{2}\rangle }\left(N_{max}\right)$ $\left[d_{conf}=0\right]$
(a)	0.40	0.524	1	11.62	9.40	10.92 (0.40); 11.00 (0.52)
(b)	0.40	0.552	0.6	10.01	7.30	10.92 (0.40); 11.01 (0.55)
(c)	0.40	0.740	0.1	5.39	2.30	10.92 (0.40);
(d)	0.05	0.057	1	23.25	10.61	10.61 (0.05)
(e)	0.20	0.247	1	14.64	10.63	10.68 (0.20); 10.77 (0.25)
(f)	0.35	0.453	1	12.16	10.57	10.90 (0.35); 10.98 (0.45)

## Data Availability

The final configurations of each computer simulation presented here are available as electronic files in the Zenodo platform through this link: https://doi.org/10.5281/zenodo.15619914.
